# Early prediction of functional prognosis in neurofibromatosis type 2 patients based on genotype–phenotype correlation with targeted deep sequencing

**DOI:** 10.1038/s41598-022-13580-9

**Published:** 2022-06-09

**Authors:** Yu Teranishi, Satoru Miyawaki, Hirofumi Nakatomi, Kenta Ohara, Hiroki Hongo, Shogo Dofuku, Atsushi Okano, Shunsaku Takayanagi, Takahiro Ota, Jun Yoshimura, Wei Qu, Jun Mitsui, Shinichi Morishita, Shoji Tsuji, Nobuhito Saito

**Affiliations:** 1 Department of Neurosurgery, Faculty of Medicine, The University of Tokyo, 7-3-1 Hongo, Bunkyo-ku, Tokyo, 113-8655 Japan; 2 Department of Neurosurgery, Faculty of Medicine, Kyorin University, Tokyo, Japan; 3 Department of Neurosurgery, Tokyo Metropolitan Tama Medical Center, Fuchu, Tokyo Japan; 4 Department of Computational Biology and Medical Sciences, Graduate School of Frontier Sciences, The University of Tokyo, Tokyo, Japan; 5 Department of Molecular Neurology, Graduate School of Medicine, The University of Tokyo, Tokyo, Japan

**Keywords:** Neurology, Signs and symptoms, Neurological disorders

## Abstract

Regardless of treatment, the clinical progression of neurofibromatosis type 2 (NF2), particularly in terms of hearing, swallowing, and gait, tend to worsen throughout the patients’ lives. We performed a retrospective analysis of functional outcomes in Japanese NF2 patients to predict their functional prognosis. We analyzed genotype–phenotype correlation based on genetic data from a cohort of 57 patients with a mean follow-up of 14.5 ± 6.0 years. Their functional outcomes, including hearing, swallowing, and ambulation, were reviewed. Performing a targeted deep sequencing, germline *NF2* mutations were identified in 28 patients (49.1%), and mosaic NF2 was identified in 20 patients (20, 35.0%). The functional preservation period and outcome differed significantly depending on clinical/genetic factors. Among these factors, “Truncating”, “Mosaic”, and “Age of symptom onset ≥ 25” had the most significant effects on functional disability. By applying a combination of an *NF2* mutation type/location, and age of symptom onset, we classified different degrees of functional preservation and progression, schwannoma growth rate and total interventions per year per patient. The prediction of detailed functional outcomes in NF2 patients can plan better strategies for life-long disease management and social integration.

## Introduction

Neurofibromatosis type 2 (NF2) is a tumour predisposition syndrome with the autosomal-dominant transmission, complete penetrance, and variable phenotypic expression caused by germline alterations in the tumour suppressor gene *NF2*^1–3^. Patients with NF2 develop uni- or bilateral vestibular schwannomas (UVS, BVS), intracranial and/or multiple spinal schwannomas, as well as multiple meningiomas^1^. The clinical diagnosis of NF2 is based on the Manchester criteria^4^, which are (1) BVS, or (2) family history and UVS, or (3) family history or UVS and two of: meningioma, cataract, glioma, neurofibroma, schwannoma, cerebral calcification (if UVS +  ≥ 2 schwannomas only need negative LZTR1 test), or 4) multiple meningioma (2 or more) and two of: UVS, cataract, glioma, neurofibroma, schwannoma, cerebral calcification, and or (5) Constitutional pathogenic NF2 gene variant in blood or identical in two tumours (Supplementary Table 1).

Patients commonly experience hearing loss, swallowing difficulties, and gait abnormalities, with many interventions required to control the progression of numerous tumors^1–3^, significantly interfering with their daily life. Despite the availability of systematic and multimodal treatments, the symptoms associated with NF2 tend to worsen throughout the patients’ lives^1–3^.

Several retrospective studies on NF2 have reported genotype–phenotype correlations as predictors of disease progression and mortality^5–11^. The resulting clinical predictors have included age of symptom onset and the presence of intracranial meningioma^5,7,12^; whereas, the genetic predictions of mortality included truncating and splicing mutations at the 5'-end of the NF2 gene^5,6,11^. Missense mutations, somatic mosaicism, and splicing at the 3'-end of the NF2 gene appeared to have the opposite effect^5,6,13^. Recently, Halliday et al. proposed a genetic severity score with germline mutation types and locations to predict mortality based on the number of tumours and clinical interventions^14^.

However, it is not only longevity but also the quality of life (QOL) and activities of daily living (ADL) with persisting symptoms that must be considered when considering development treatment strategies. To date, a limited number of studies have reported genotype–phenotype correlations as predictors of detailed functional prognosis in NF2 patients^15–17^. Moreover, the number of articles about NF2 in the Asian population is also limited^12,18–21^. Thus, to improve the clinical care of NF2 patients and early predict their functional prognosis, we used retrospective genetic analysis with target deep sequencing focused on the functional outcomes of Japanese NF2 patients.

## Results

### Clinical characteristics

All 57 patients (24 men and 33 women) whose blood samples were obtained fulfilled the Manchester Clinical Diagnostic Criteria for NF2^4^. Age of symptom onset ranged from 4 to 61 years (mean 25.5 ± 13.3) (Table 1). Specific lesions were as follows: vestibular schwannoma (bilateral: 84.2%, unilateral: 15.7%), intracranial meningiomas (56.1%), other cranial nerve schwannomas (47.3%), spinal schwannoma (64.9%), spinal ependymoma (22.8%), ocular lesion (22.8%), and cutaneous lesions (50.8%) (Table 1). Only four patients (7%) had a family history of NF2.

**Table 1:** Clinical characteristics of NF2 patients.

	Total	Truncating	Large deletion	Splice site	Missense	Mosaic	Undetected
Patients (n)	57	12	4	9	3	20	9
Years of follow up (years; mean ± SD)	14.5 ± 6.0	14.3 ± 5.7	13.5 ± 3.6	18.1 ± 7.0	15.3 ± 4.9	13.1 ± 4.8	14.6 ± 8.6
Family history of NF2 ( +) n (%)	4 (7)	0 (0)	1(25)	2 (22.2)	1(33.3)	0 (0)	0 (0)
Vital status: alive n (%)	55 (96.5)	10 (83.3)	4 (100)	9 (100)	3 (100)	20 (100)	9 (100)
Sex n (%)							
Male	24 (42.1)	6 (50)	2 (50)	6 (66.7)	0 (0)	7 (35)	3 (33.3)
Female	33(57.9)	6 (50)	2(50)	3(33.3)	3(100)	13(65)	6(66.7)
Age of symptom onset (mean ± sd)	25.5 ± 13.3	13.1 ± 6.0	21.5 ± 5.0	22.2 ± 8.9	27.0 ± 2.6	33.6 ± 14.2	28.7 ± 14.1
Bilateral vestibular schwannoma n (%)	48 (84.2)	12 (100)	4 (100)	9 (100)	3 (100)	14 (70)	6 (66.7)
Intracranial meningioma n (%)	32 (56.1)	8 (66.7)	1 (25)	6 (66.7)	1 (33.3)	10 (50)	6 (66.7)
Cranial nerve Schwannoma n (%)	27 (47.4)	9 (66.7)	3 (75)	8 (88.9)	1 (33.3)	3 (15)	3 (33.3)
Spinal Schwannoma n (%)	37 (65)	11 (91.7)	3 (75)	8 (88.9)	2 (66.7)	8 (40)	5 (55.6)
Spinal Ependymoma n (%)	13 (22.8)	5 (41.7)	2 (50)	3 (33.3)	0 (0)	3 (15)	0 (0)
Ocular lesion n (%)	13 (22.8)	4 (33.3)	3 (75)	3 (33.3)	0 (0)	1 (5)	2 (22.2)
Cutaneous lesion n (%)	29 (50.9)	12 (100)	4 (100)	9 (100)	3 (100)	1 (5)	0 (0)
Total interventions/pt/year(n; mean ± sd)	0.2 ± 0.2	0.4 ± 0.3	0.1 ± 0.05	0.3 ± 0.3	0.3 ± 0.1	0.1 ± 0.09	0.1 ± 0.1
Cranial surgery/pt/year (n; mean ± sd)	0.1 ± 0.1	0.2 ± 0.2	0.04 ± 0.05	0.2 ± 0.2	0.1 ± 0.08	0.1 ± 0.07	0.1 ± 0.09
Spinal surgery/pt/year (n; mean ± sd)	0.05 ± 0.1	0.1 ± 0.2	0.01 ± 0.02	0.06 ± 0.09	0.01 ± 0.02	0.02 ± 0.03	0.03 ± 0.07
Radiation therapy/pt/year (n; mean ± sd)	0.04 ± 0.06	0.07 ± 0.07	0.07 ± 0.09	0.05 ± 0.06	0.1 ± 0.1	0.01 ± 0.03	0.004 ± 0.01

### Functional/survival outcomes

Among the 57 patients, the Karnofsky Performance Scale (KPS) score of 18 (31.5%) patients were less than 40. Although 31 patients (54.3%) maintained useful hearing, the rest already had disabled hearing or deafness at presentation. Regarding swallowing function, 14 patients (24.5%) already presented “Tube feeding.” Lastly, 17 patients (29.8%) already showed “Wheelchair-bound or bedridden” in gait function. Fifty-five patients were alive at the end of 2019.

### Mutation analysis

Germline *NF2* mutations were identified in 28 patients (49.1%), including truncating mutations (12, 21.0%), large deletions (4, 7.0%), splice site mutations (9, 15.7%), and missense mutations (3, 5.2%). Mosaic NF2 was identified in 20 patients (35.0%) (mosaic truncating mutation (16, 28.0%), mosaic in–frame deletion (1, 1.7%), and mosaic missense mutations (3, 5.2%) (Tables 1, 2; Supplementary Table 2).

**Table 2:** Genotype characteristics of NF2 patients.

Pt	Sex	Onset age	Follow-up	Germline/Mosaicism	VAF (%)	*NF2* variant	Amino acid	Exon	Mutation type	*NF2* variant in tumor
1	M	14	5	G	#	c.1021C>T	p.Arg341*	Ex11	Nonsense	c.1021C>T
2	M	14	14	G	#	c.1084C>T	p.Gln362*	Ex11	Nonsense	c.1084C>T
3	F	5	21	G	#	c.1345A>T	p.Lys449*	Ex13	Nonsense	c.1345A >T
4	M	9	11	G	#	c.1526delT	p.Glu509Sfs*6	Ex14	Fr. del	Unavailable
5	F	22	21	G	#	c.140_141del	p.Phe47*	Ex2	Fr. del	Unavailable
6	M	9	7	G	#	c.169C>T	p.Arg57*	Ex2	Nonsense	c.169C>T
7	F	4	16	G	#	c.580delG	p.Glu194Sfs*15	Ex6	Fr. del	c.580delG
8	M	16	20	G	#	c.572G>A	p.Trp191*	Ex6	Nonsense	c.572G>A
9	M	24	9	G	#	c.592C>T	p.Arg198*	Ex6	Nonsense	Unavailable
10	F	13	22	G	#	c.634C>T	p.Gln212*	Ex7	Nonsense	c.634C>T
11	F	12	12	G	#	c.784C>T	p.Arg262*	Ex8	Nonsense	c.784C>T
12	F	16	14	G	#	c.213delA	p.Val72Wfs*51	Ex2	Fr. del	c.213delA
13	F	15	19	G	#	Exon deletion		Ex.1–6	Large del	Unavailable
14	M	20	12	G	#	Exon deletion		Ex.2–16	Large del	Unavailable
15	F	26	12	G	#	Exon deletion		Ex.15	Large del	Unavailable
16	M	26	11	G	#	Exon deletion		Ex.1–16	Large del	Ex.1–16 deletion
17	M	20	17	G	#	c.1447-20_1447-3del		Ex14	Splice site	c.1447-20_1447-3del
18	M	23	11	G	#	c.363 + 1G>A		Ex3	Splice site	c.363 + 1G >A
19	M	14	8	G	#	c.1122 + 2 T>C		Ex11	Splice site	Unavailable
20	F	12	27	G	#	c.1446 + 1G>C		Ex13	Splice site	Unavailable
21	M	12	27	G	#	c.1341-2A>G		Ex13	Splice site	Unavailable
22	M	23	12	G	#	c.1575-1G>A		Ex15	Splice site	c.1575-1G>A
23	M	37	21	G	#	c.1575-1G>A		Ex15	Splice site	c.1575-1G>A
24	F	26	16	G	#	c.448-2A>T		Ex5	Splice site	c.448-2A>T
25	F	33	24	G	#	c.517-2A>G		Ex6	Splice site	Unavailable
26	F	29	12	G	#	c.1340G>A	p.Arg447Lys	Ex12	Missense	c.1340G>A
27	F	24	13	G	#	c.239A>G	p.Lys80Arg	Ex2	Missense	c.239A>G
28	F	28	21	G	#	c.137 T>A	p.Leu46His	Ex2	Missense	c.137 T>A
29	M	22	9	M	11	c.1611_1651del	p.Glu537Aspfs*14	Ex15	Nonsense	Unavailable
30	F	25	22	M	10	c.169C>T	p.Arg57*	Ex2	In-fr. del	c.169C>T
31	F	21	19	M	10	c.286_288del	p.Phe96del	Ex3	Fr. del	c.286_288del
32	M	11	14	M	9.4	c.1396C>T	p.Arg466*	Ex13	Nonsense	Unavailable
33	M	46	15	M	9.1	c.1366C>T	p.Gln456*	Ex13	Nonsense	c.1366C>T
34	F	32	17	M	7.2	c.1174G>T	p.Glu392*	Ex12	Nonsense	Unavailable
35	F	19	17	M	5.2	c.36_37del	p.Leu14Glnfs*34	Ex1	Fr. del	Unavailable
36	M	61	13	M	4.0	c.592C>T	p.Arg198*	Ex6	Nonsense	c.592C > T
37	F	56	19	M	2.8	c.586C>T	p.Arg196*	Ex6	Nonsense	c.586C > T
38	M	33	4	M	2.1	c.1366C>T	p.Gln456*	Ex13	Nonsense	Unavailable
39	M	33	13	M	1.7	c.784C>T	p.Arg262*	Ex8	Nonsense	c.784C>T
40	F	17	10	M	1.4	c.439C>A	p.Gln147Lys	Ex4	Missense	Unavailable
41	F	39	8	M	1.0	c.361C>T	p.Gln121*	Ex3	Nonsense	c.361C>T
42	F	37	8	M	0.9	c.334G>T	p.Glu112*	Ex3	Nonsense	Unavailable
43	F	45	15	M	0.9	c.1396C>T	p.Arg466*	Ex13	Nonsense	Unavailable
44	F	60	11	M	0.8	c.1766G>A	p.Cys589Tyr	Ex16	Missense	Unavailable
45	M	30	18	M	0.8	c.293_303del	p.Lys99*	Ex3	Fr. del	Undetected
46	F	22	14	M	0.7	c.1439C >T	p.Thr480Met	Ex13	Missense	Unavailable
47	F	29	11	M	0.5	c.773G>A	p.Trp258*	Ex8	Nonsense	c.773G>A
48	F	36	5	M	0.1	c.892C>T	p.Gln298*	Ex10	Nonsense	c.892C>T
49	F	16	6	Undetected						Unavailable
50	F	21	24	Undetected						Unavailable
51	M	25	23	Undetected						Unavailable
52	M	24	23	Undetected						Undetected
53	F	17	22	Undetected						Unavailable
54	F	59	5	Undetected						Unavailable
55	F	32	8	Undetected						Undetected
56	F	22	5	Undetected						Undetected
57	M	43	15	Undetected						Unavailable

VAF, variant allele frequency; G, germline; M, mosaicism; Fr., frameshift; Del., deletion, #, we detected germline *NF2* alteration with Sanger sequence and MLPA.

### Genotype–phenotype correlation using the Kaplan–Meier method and univariate/multivariate Cox proportional hazards model

**Figure 1:**
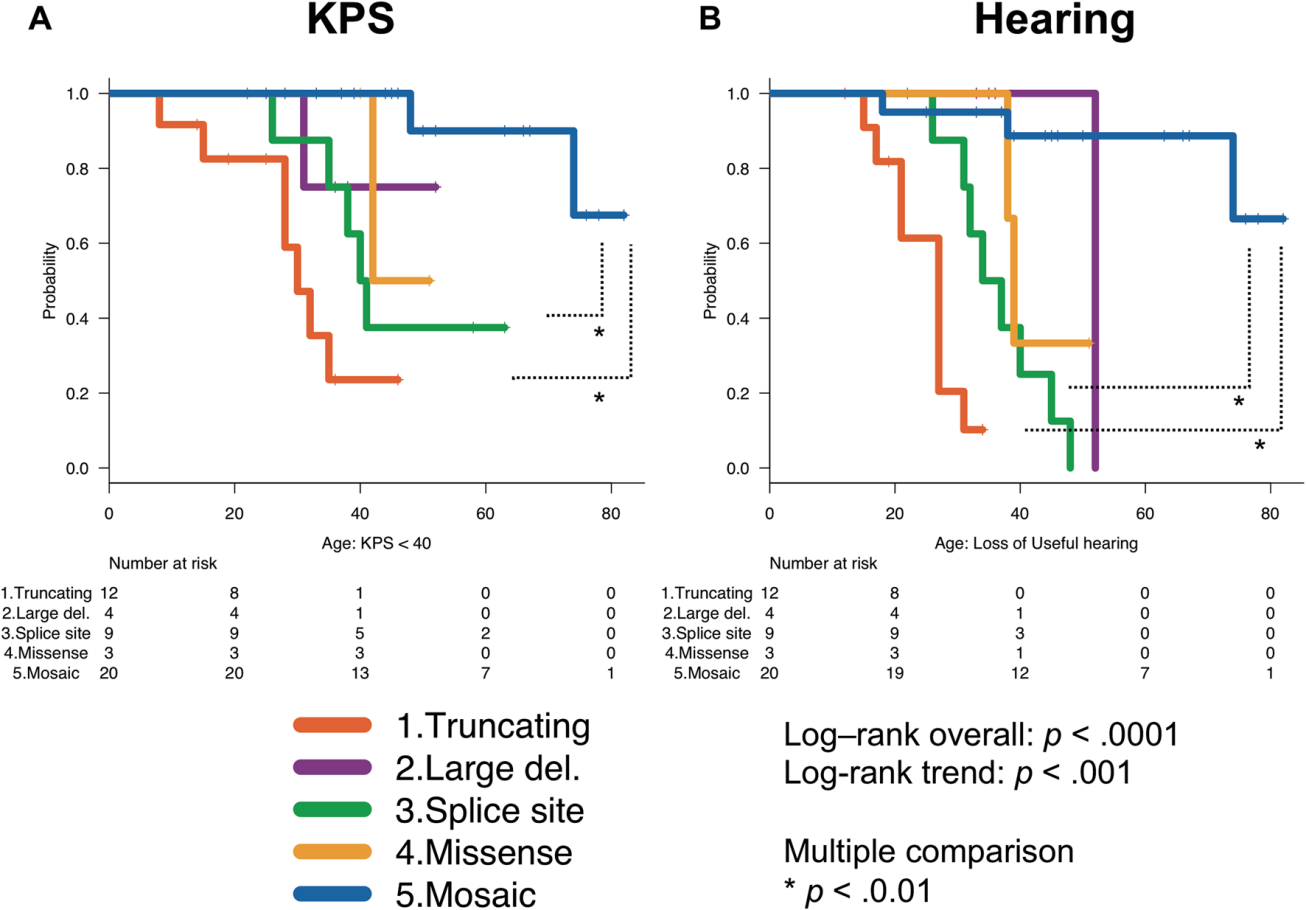
Kaplan–Meier curves of five *NF2* mutation types (“Truncating”, “Large deletion”, “Splice site”, “Missense”, “Mosaic”) with the functional outcome (A: KPS, B: hearing) in NF2 patients (statistical analysis and significance are detailed in the main text).

The periods of functional outcome preservation differed statistically significantly (log-rank test: p < 0.001) depending on the type of *NF2* mutation (Fig. 1; supplementary Fig. 1). Univariate and multivariate Cox proportional hazards models examining the effects of all clinical/genetic factors on functional disability were performed for all patients (Supplementary table 3–6). “Truncating” (HR 3.97, 95% CI 1.28–12.25, p = 0.016) and “Mosaic” (HR 0.09, 95% CI 0.01–0.76, P = 0.027) had significant effects on KPS ≤ 40 (Supplementary table 3). For “disabled hearing or deafness”, “Truncating” (HR 11.61, 95% CI 1.77–75.85, p = 0.01), “Splice site” (HR 5.65, 95% CI 1.02–31.14, p = 0.046) and “Age of symptom onset ≥ 25” (HR 0.01, 95% CI 0.0006–0.17, p = 0.001) were significant predictors (Supplementary table 4).

### Progression of functional disability and schwannoma growth rate

**Figure 2:**
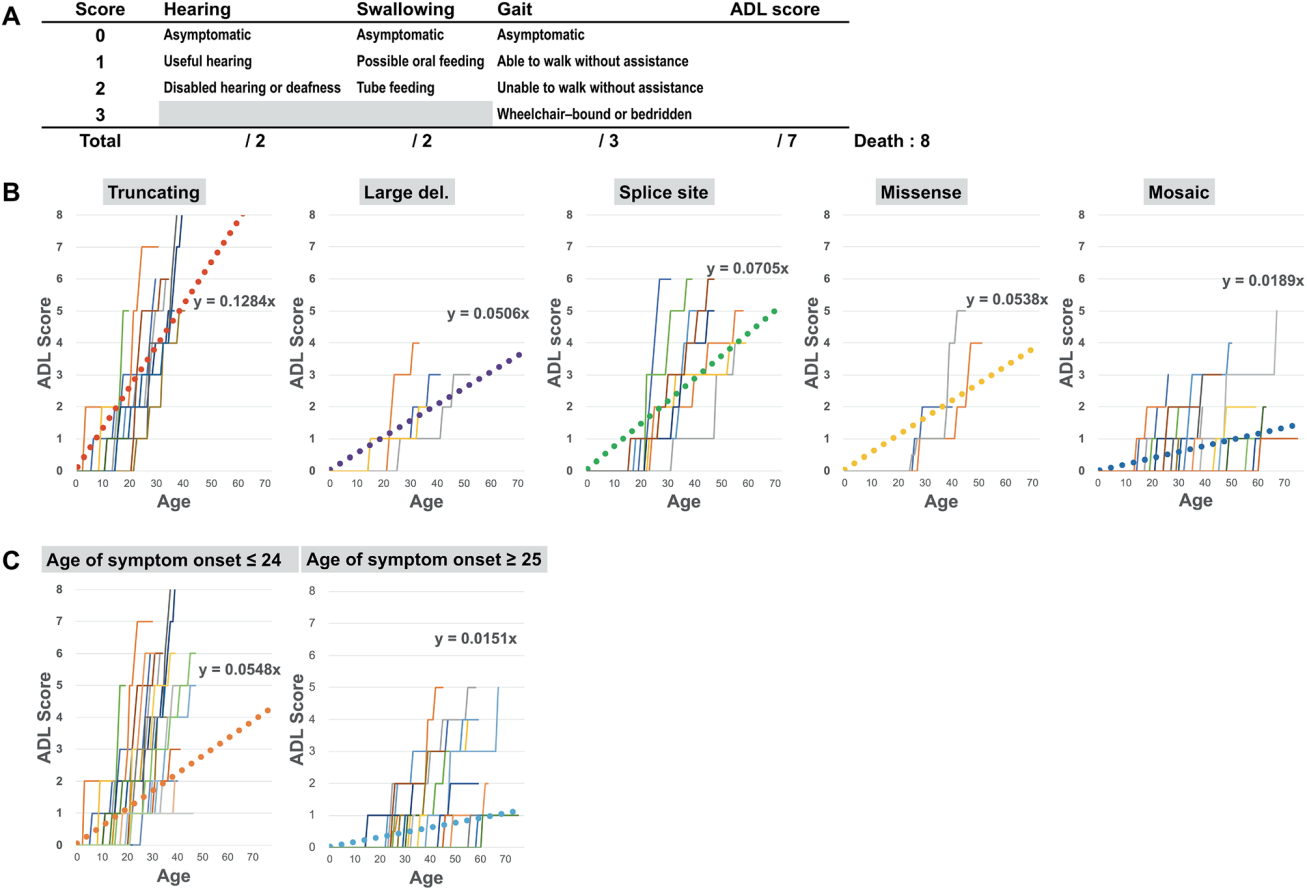
Progression of the functional disability (ADL score) of NF2 patients according to each of the five *NF2* mutation types (“Truncating”, “Large deletion”, “Splice site”, “Missense”, “Mosaic”) and age of symptom onset. To evaluate the progression of functional disability of each NF2 patient, we registered the total ADL score (0–7, death = 8) of each against age and created line graphs plotting the progression of functional disability throughout their life (x-axis: age, y-axis: total ADL score). Dotted line: average of gradients, denoting the speed of ADL progression using a linear functional approximate expression of the line graph.

The line plotting the progression of functional disability throughout each patient’s life (Fig. 2) showed significant differences for each mutation type and age of symptom onset. The difference was evident by statistically comparing the line plots’ gradients (Supplementary Fig. 2). The schwannoma growth rate was also found to differ significantly depending on the type of mutation and “Age of symptom onset ≥ 25” (Supplementary Fig. 2).

### Our classification by applying a combination of an NF2 mutation type/location, and age of symptom onset

**Figure 3:**
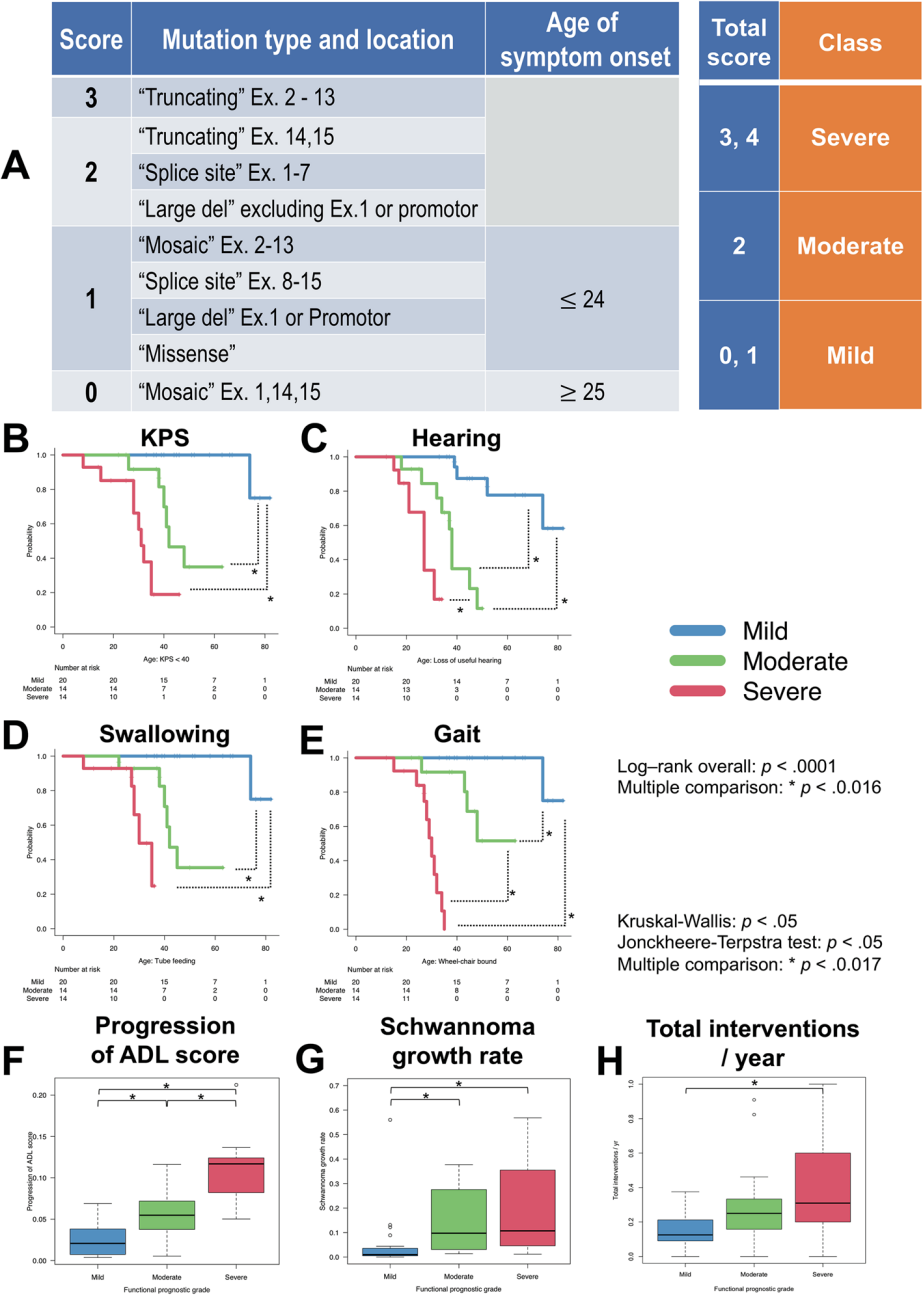
NF2 functional prognostic grade at diagnosis. 3A: The NF2 functional prognostic grading scale classified patients into three grades (mild, moderate, and severe) based on the *NF2* mutation types, *NF2* mutation location, and age of symptom onset (statistical analysis and significance are detailed in the main text). 3B-3E: Kaplan–Meier curves of each functional prognostic grade for KPS, hearing, swallowing, and gait. 3F-3H: A boxplot depending on each functional prognostic grade shows the relationship between ADL score progression, schwannoma growth rate, and total interventions/year. This grading system indicates the different functional disabilities and the different biological tumour behaviours for each grade.

According to Halliday’s genetic severity score^14^ and our results, we classified our cohort into three classes (mild, moderate, or severe) based on the *NF2* mutation types, *NF2* mutation location, and age of symptom onset (Fig. 3). Comparing the three classes, each class (mild, moderate, severe) was significantly different, with shorter or longer periods of functional preservation for each type of functional disability. There was also a significant difference among the gradients to the progression of the ADL score, the schwannoma growth rate, and total interventions/year (Fig. 3).

**Figure 4:**
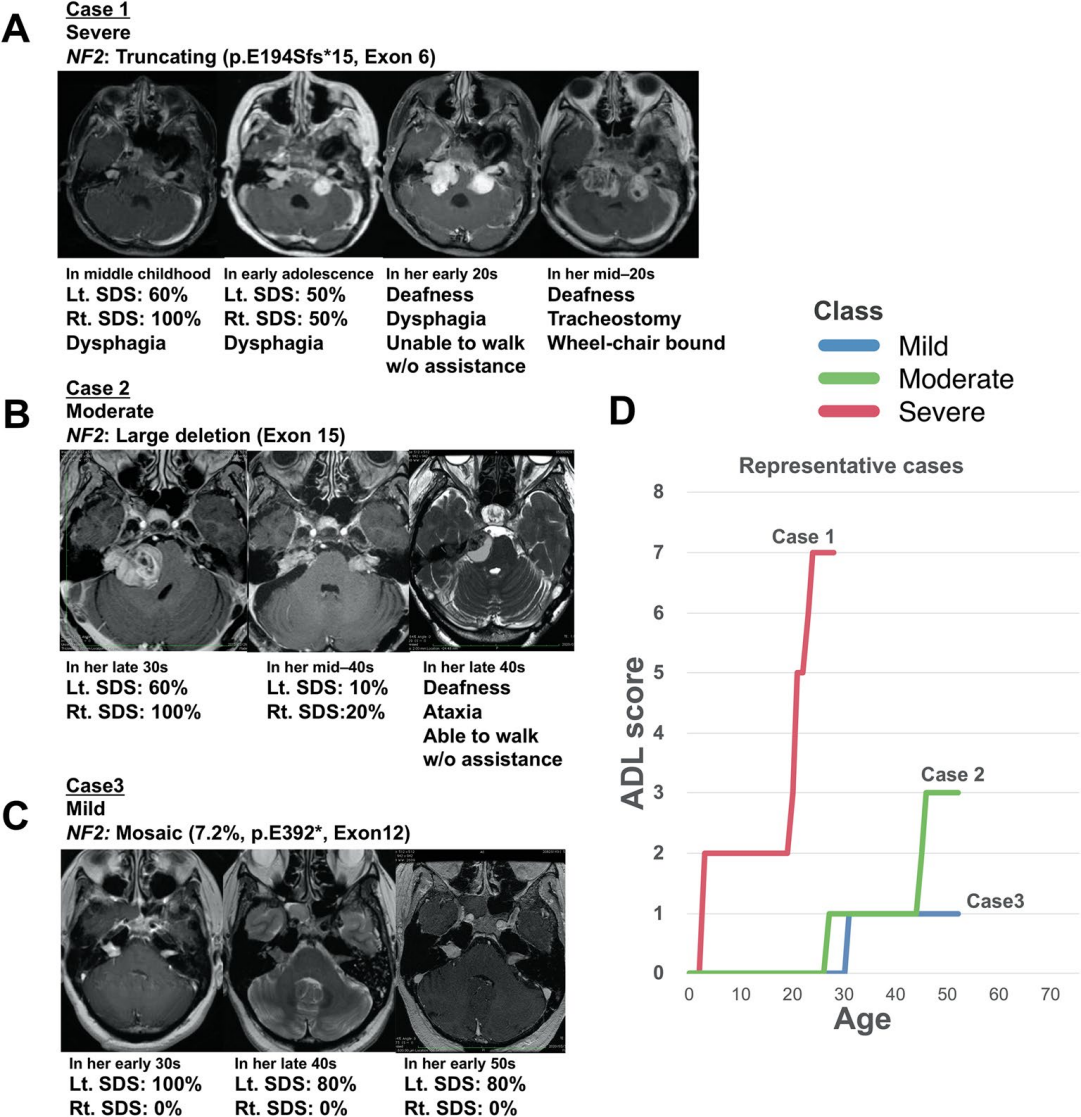
Representative cases of NF2 functional prognostic grading system. Case 1: NF2 functional prognostic grade “severe” (Truncating: p.Glu194Sfs*15, Exon 6, age of symptom onset = in middle childhood). Case 2: NF2 functional prognostic grade “moderate” (Large deletion, exon 15, age of symptom onset = in her mid-20 s). Case 3: NF2 functional prognostic grade “mild” (mosaic: VAF 7.2%, p.Glu392*, exon 12, age of symptom onset = in her early 30 s).

Representative cases diagnosed using Manchester’s NF2 criteria are shown in Fig. 4. Case 1 was classified as “severe” (Fig. 4, case 1). In middle childhood, she underwent left meningioma surgery, presenting dysphagia and hoarseness in another hospital. During early adolescence, the patient was referred to us. Her MRI showed bilateral vestibular schwannomas, bilateral trigeminal schwannomas (Lt. SDS: 60%, Rt. SDS: 100%). Afterwards, the annual follow-up indicated gradual hearing deterioration (Lt. SDS: 50%, Rt. SDS: 50%). In her early 20 s, she finally lost hearing function. Furthermore, the patient developed severe dysphagia and gait disturbance. Eventually, she underwent a tracheostomy and became wheelchair-bound. Since the age of disease onset, she underwent 17 interventions, including tumour resections, radiosurgery, ventriculoperitoneal shunt, tracheostomy, and abscess removal.

Case 2 was classified as “moderate” (Fig. 4, case 2). She had a family history of NF2. In her late 30 s, she was referred to us for right vestibular schwannoma surgery, with already impaired right hearing function (Lt. SDS: 60%, Rt. SDS: 100%). After tumour resection, the annual follow-up indicated gradual hearing deterioration. In her mid–40 s, the patient finally presented a bilateral loss of useful hearing (Lt. SDS: 10%, Rt. SDS: 20%). At present, the patient presents gait disturbance due to brain stem compression due to a growing right trigeminal schwannoma.

Case 3 was classified as “mild” (Fig. 4, case 3). She underwent resection of a right vestibular schwannoma with initial hearing impairment in her early 30 s. After tumour resection, she lost right useful hearing (Lt. SDS: 100%, Rt. SDS: 0%). In her early 40 s, she has referred again for recurrence of the operated tumour treated with radiosurgery, but she had stable left useful hearing (Lt. SDS: 80%, Rt. SDS: 0%). In her early 50 s, the patient had useful hearing with stable schwannomas on the yearly MRI (Lt. SDS: 80%, Rt. SDS: 0%).

## Discussion

Given recent improvements in these patients’ prognoses due to the development of more effective treatment procedures^5^, the issues of ADL maintenance and the effect of persisting symptoms on patient QOL are becoming increasingly important. We analyzed the clinical and genetic predictors of the functional disability of NF2 patients. Two types of mutations were found to act as predictors, namely “Truncating” and “Mosaic”, in addition to “Age of symptom onset ≥ 25”. Consequently, we showed that patients of “Mild” had different functional prognoses compared with other NF2 patients (Figs. 3, 4).

In terms of the prediction of outcomes in NF2 patients, Halliday’s genetic severity score based on the type and location of germline mutations has been known^14^. Characteristically, their genetic severity score included site and degree in not only germline *NF2* alteration but also mosaicism^14^. For clinical use, they suggested three groups: “1: Tissue mosaic,” “2: Classic,” and “3: Severe”^14^. In particular, their genetic severity score distinguished mosaic in the blood (subcategory: 2A(Mild), 2B(Moderate)) from tissue mosaic (subcategory: 1B)^14^. However, when Halliday’s genetic severity score was applied in our cases, it did not work well (Supplementary Fig. 3). In particular, the Kaplan–Meier curves for functional disabilities showed that subcategory 2A was worse than the 2B group subcategory (Supplementary Fig. 3). Furthermore, the latest report showed no significant survival difference between 2A(Mild) and 2B(Moderate) in Halliday’s genetic severity score^22^. We performed targeted deep sequencing for multiple tissue DNA to detect a low variant allele frequency (VAF) *NF2* variant. Many mosaic NF2 in our cases, which are assumed to include subcategory 1B in previous genetic severity score, were included in subcategory 2B because most mosaic cases were detected in exon 2–13 (2A: exon 14,15 mosaic, 2B: exon 2–13 mosaic)^14^. Consequently, the Kaplan–Meier analysis showed that the functional outcome of group 2B was milder than that of group 2A. Considering this situation and our results, we emphasized the meaning of “Truncating” as a predictor for worsening functional outcomes and the importance of “Mosaic” as a predictor for slower functional disability.

Nine patients present without any *NF2* alteration (Supplementary Table 7). Interestingly, some patients of these nine patients present with a similar phenotype to that of the patient with a pathogenic *NF2* variant. Others also show the older onset age, UVS and multiple schwannomas (they have no mutation with *LZTR1* by WES). We have already performed Sanger sequence, MLPA, whole exome sequence, and targeted deep sequencing for these nine patients, but we could not identify any germline or low variant frequency *NF2* alteration, *LZTR1*, and *SMARCB1* mutation. We will perform whole-genome sequences and long-read sequences in future.

This was a retrospective study with a relatively small number of enrolled NF2 patients (n = 57). And the relatively homogeneous genetic background may influence our evaluation. Our classification did not include the degree of mosaicism compared with Halliday’s genetic severity score. Hence, more patients from abroad should be recruited in the future to assess the clinical utility of our classification considering the degree of mosaicism. We thought that our main characteristics of this study were “Asian cohort”, “high rate of detected mosaic NF2 with targeted deep sequencing”, and “detailed functional outcome”.

Even if we performed mutational analysis of multiple normal tissue DNA to compensate for limited read depth, distinguishing between a true variant and error could be still problematic. Furthermore, the phenotype of mosaic cases is known as highly variable^23^. Identified mosaic cases are more severely affected and more likely to have presented with classical bilateral VS^23^. Considering the variation of the phenotype in mosaic NF2 and the accuracy of detecting mutation, diagnosing and utilising mosaic NF2 remains a limitation.

This study performed genotype–phenotype correlation analysis for the early prediction of functional prognosis in Japanese NF2 patients. By applying a combination of an *NF2* mutation type/location, and age of symptom onset, we classified different degrees of functional preservation and progression, schwannoma growth rate and total interventions per year per patient. The prediction of detailed functional outcomes can be used to plan better strategies for life-long disease management and social integration.

## Materials and methods

All methods were carried out in accordance with relevant guidelines and regulations.

### Patient population

The data of eighty-five patients with an established diagnosis of NF2 according to Manchester’s NF2 criteria^4^ at The University of Tokyo Hospital and the Tokyo Metropolitan Tama Medical Center from 2000 to 2019 were used in our analysis. We retrospectively analyzed the clinical data, imaging studies, operative findings, and functional outcomes obtained from our hospital clinical database and existing medical records. We performed a patient interview directly in the outpatient clinic (37 patients) or by phone (20 patients). Twenty-eight patients were excluded because of incomplete clinical data. The remaining 57 patients attended our outpatient clinics at least once a year and performed the diagnostic and treatment procedures when required. These 57 patients included 53 de novo NF2 patients reported in our previous study^21^. The average follow-up period was 14.5 ± 6.0 years. All patients underwent complete neurological evaluation and hearing tests, including audiogram before and after surgery and at each follow-up visit. Imaging evaluation was performed with MRI preoperatively and postoperatively and annually thereafter.

### Mutation analysis and bioinformatics analysis

DNA of NF2 patients was obtained from their peripheral blood leucocytes, buccal swabs, hair follicles, and tumour samples. As previously described, mutation analysis of *NF2* (GenBank accession number, NM_000268.4) was performed, including direct Sanger sequencing, multiple ligation-dependent probe amplification (MLPA), and target deep next-generation sequencing. Sequencing reads were aligned to the human genome (hg19).

As mosaic NF2 has been previously defined (supplementary Table 1)^21^, proven mosaic NF2 was considered confirmed when *NF2* variant with low VAF was detectable (1) in blood, (2) in both buccal and hair DNA but not in blood, and (3) detection of *NF2* variant in two independent tumour DNA samples. Data analysis of the target deep sequence was performed as previously described^21^.

### Evaluation of functional disability progression in NF2 patients

Functional disability in the NF2 patients was evaluated using KPS score and our new ADL scoring system (Fig. 2). This system was composed of three major functional scales, based on “hearing” (score 0–2), “swallowing” (score 0–2), and “gait” (score 0–3). Assessments were performed in patient interviews, establishing when and how these major functions were impaired, either directly in the outpatient clinic (37 patients) or by phone (20 patients). “Hearing” was classified as “asymptomatic” (Gardner-Robertson Hearing Scale Grade I) (score: 0), “useful hearing” (Gardner-Robertson Hearing Scale Grade II) (score: 1), “disabled hearing (Gardner-Robertson Hearing Scale Grade III, IV, V) or deafness” (score: 2). We classified “swallowing” into “asymptomatic” (score: 0), “possible oral feeding (a patient report of some difficulty with swallowing sufficient)” (score: 1), and “tube feeding” (score: 2). “Gait” was scored as “asymptomatic” (score: 0), “able to walk without assistance (a patient report of some difficulty with walking sufficient)” (score: 1), “able to walk with assistance” (score: 2), and “Wheelchair-bound or bedridden” (score: 3). To evaluate the progression of functional disability in each NF2 patient, we registered the total ADL score (0–7, death = 8) against age and created line-plotted graphs expressing the progression of functional disability throughout their life (x-axis: age, y-axis: total ADL score) (Fig. 2). Furthermore, we calculated a gradient expressing ADL progression speed using a linear functional approximate expression of the line plot graph and compared each NF2 patient gradient to genetic and clinical factors (Fig. 3).

### The growth rate of schwannoma and meningioma

Tumour volumetric analysis was performed as previously described using the volumetric function of the OsiriX Lite ver. 9.0 software^21^. We measured the total volume of intracranial schwannoma and meningioma for each patient, respectively and compared each total volume in the initial MRI studies with those of the last study to calculate the tumour growth rate.

### Total interventions per year

By reviewing the medical records and our clinical database, we calculated the number of interventions each NF2 patient underwent during the follow-up period, including surgical treatment and radiotherapy for primary or recurrent tumours. The number of interventions for each patient was then divided by the follow-up duration (in years).

### Statistical analysis

Statistical analyses were performed using the survival and ggplot2 packages in R v.3.1.2 (http://www.R-project.org).

Categorical variables were expressed as the mean and standard deviation (SD). The ADL outcomes, namely the period of functional preservation, were estimated for each mutation group and each prognostic grade using the Kaplan–Meier method, followed by the log-rank test to determine the total and trends. Each curve was compared using the log-rank test after Bonferroni correction.

The hazard ratio of all clinical/genetic factors for all major functional endpoints (“Disabled hearing or deafness,” “Tube feeding,” and “Wheelchair-bound or bedridden”) was analyzed using the univariate Cox proportional hazards model. Variables associated with functional endpoints in univariate analyses (P < 0.05) were included in a backward, stepwise Akaike’s Information Criterion (AIC) multivariate analysis. We checked the proportional hazards assumption for each variable by testing Schoenfeld residuals and using the double-log plot method.

Clinical factors included age of symptom onset (“25 ≦” or “24 ≧”; based on the mean of age of symptom onset), onset symptoms (hearing impairment or not), presence of an extra-CNS lesion, presence of intracranial meningioma, and family history. Genetic factors included types of germline mutations (“Truncating”, “Large deletion”, “Splice site”, “Missense” and “Mosaic”), and the locations of the germline mutations according to the functional domains (exon 2–7, exon 8–13, and exon 1 and 14–15).

The Mann–Whitney U test was used to compare two non-parametric continuous variables, while the Kruskal–Wallis and Steel–Dwass tests were used for multiple comparisons with non-parametric continuous variables. The Jonckheere-Terpstra test was used to test the trend across the five mutation groups and the four prognostic grades.

All reported *p*-values are two-sided. In all comparisons, *p-*values of less than 0.05 were considered statistically significant. Post-hoc pairwise comparisons between the five mutation groups or the four prognostic grades were adjusted using the Bonferroni method.

### Ethical approval

The Institutional Review Board approved the study protocol of the University of Tokyo Hospital (G10026), and informed consent was obtained from all patients.

## Supplementary Information

Supplementary Information.

## Data Availability

The authors confirm that the data supporting the findings of this study will be shared by request from any qualified investigator.
