# Supplementary material for: Early prediction of functional prognosis in neurofibromatosis type 2 patients based on genotype–phenotype correlation with targeted deep sequencing
Source: Sci Rep. 2022 Jun 9;12:9543. doi: 10.1038/s41598-022-13580-9 (PMC9184572; doi:10.1038/s41598-022-13580-9)
Supplement: Supplementary file 1 — Supplementary Information. [file 41598_2022_13580_MOESM1_ESM.pdf]

Supplementary Table 1. The Manchester Criteria for NF2

1. Bilateral vestibular schwannomas OR

2. Family history AND unilateral VS OR

3. Family history OR unilateral VS AND two of:[a] meningioma, cataract, glioma, neurofibroma, schwannoma, cerebral calcification (if UVS + ≥2 schwannomas only need negative LZTR1 test)[b], OR

4. Multiple meningioma (2 or more) AND two of: unilateral VS, cataract, glioma, neurofibroma, schwannoma, cerebral calcification, OR

5. Constitutional pathogenic NF2 gene variant in blood or identical in two tumors[b]

NF2: neurofibromatosis 2, UVS: universal vestibular schwannoma, VS: vestibular schwannoma.

[a]Includes two of any tumor type such as schwannoma.

[b]Requires molecular analysis.

Supplementary Table 2 Detailed sequense data

Pt	*NF2* variant	*Germline or Mosaic*	Sanger seq	MLPA	WES	Targeted deep sequence	Blood VAF	Blood Mut. Reads	Blood Ref. reads	Buccal mucosa VAF	Buccal mucosa Mut. reads	Buccal mucosa Ref. reads	Hair follicle VAF	Hair follicle Mut. reads	Hair follicle Ref. reads	Tumor VAF	Tumor Mut. reads	Tumor Ref. reads
1	c.1021C>T	Germline	+	+	-	-												
2	c.1084C>T	Germline	+	+	-	-												
3	c.1345A>T	Germline	+	+	-	-												
4	c.1526delT	Germline	+	+	-	-												
5	c.140_141del	Germline	+	+	-	-												
6	c.169C>T	Germline	+	+	-	-												
7	c.580delG	Germline	+	+	-	-												
8	c.572G>A	Germline	+	+	-	-												
9	c.592C>T	Germline	+	+	-	-												
10	c.634C>T	Germline	+	+	-	-												
11	c.784C>T	Germline	+	+	-	-												
12	c.213delA	Germline	+	+	-	-												
13	Exon deletion	Germline	+	+	-	-												
14	Exon deletion	Germline	+	+	-	-						Unavailable						
15	Exon deletion	Germline	+	+	-	-												
16	Exon deletion	Germline	+	+	-	-												
17	c.1447-20_1447-3del	Germline	+	+	-	-												
18	c.363+1G>A	Germline	+	+	-	-												
19	c.1122+2T>C	Germline	+	+	-	-												
20	c.1446+1G>C	Germline	+	+	-	-												
21	c.1341-2A>G	Germline	+	+	-	-												
22	c.1575-1G>A	Germline	+	+	-	-												
23	c.1575-1G>A	Germline	+	+	-	-												
24	c.448-2A>T	Germline	+	+	-	-												
25	c.517-2A>G	Germline	+	+	-	-												
26	c.1340G>A	Germline	+	+	-	-												
27	c.239A>G	Germline	+	+	-	-												
28	c.137T>A	Germline	+	+	-	-												
29	c.1611_1651del	Mosaic	+	+	+	+		Sanger [#3]			Unavailable			Unavailable			Sanger [#1]	
30	c.169C>T	Mosaic	+	+	+	+		Sanger [#3]			Unavailable			Unavailable			Sanger [#1]	
31	c.286_288del	Mosaic	+	+	+	+	11.60%	3	26 [#2]		Unavailable			Unavailable			Unavailable	
32	c.1396C>T	Mosaic	+	+	+	+	0.07%	1	1321	-	0	174	9.41%	34	361		Unavailable	
33	c.1366C>T	Mosaic	+	+	+	+	9.09%	97	1067		Unavailable			Unavailable		90.40%	2866	3170
34	c.1174G>T	Mosaic	+	+	+	+	-	0	2076	-	0	358	7.13%	96	1346		Unavailable	
35	c.36_37del	Mosaic	+	+	+	+	5.20%	35	672		Unavailable			Unavailable			Unavailable	
36	c.592C>T	Mosaic	+	+	+	+	4.00%	1	28 [#2]		Unavailable			Unavailable		43.30%	13	30
37	c.586C>T	Mosaic	+	+	+	+	0.25%	1	395	2.83%	49	1730	-	0	257	53.30%	168	315
38	c.1366C>T	Mosaic	+	+	+	+	2.14%	12	560		Unavailable			Unavailable			Unavailable	
39	c.784C>T	Mosaic	+	+	+	+	0%	0	1526	1.62%	3	185	1.71%	16	933	43.80%	138	315
40	c.439C>A	Mosaic	+	+	+	+	1.46%	6	410	0.06%	1	1444	-	0	517		Unavailable	
41	c.361C>T	Mosaic	+	+	+	+	0.95%	4	419		Unavailable			Unavailable		56.80%	392	690
42	c.334G>T	Mosaic	+	+	+	+	0.86%	38	4405		Unavailable			Unavailable			Unavailable	
43	c.1396C>T	Mosaic	+	+	+	+	-	0	1168	0.07%	1	1352	0.86%	10	1154		Unavailable	
44	c.1766G>A	Mosaic	+	+	+	+	-	0	1767	-	0	471	0.89%	5	556		Unavailable	
45	c.293_303del	Mosaic	+	+	+	+	0.78%	14	1791		Unavailable			Unavailable			Undetected	
46	c.1439C>T	Mosaic	+	+	+	+	-	0	1645	0.73%	4	541		Unavailable			Unavailable	
47	c.773G>A	Mosaic	+	+	+	+	0.06%	1	1664	0.10%	1	661	0.58%	4	689	64.40%	1134	1760
48	c.892C>T	Mosaic	+	+	+	+	0.14%	3	2042		Unavailable			Unavailable			Sanger [#1]	
49	Undetected		+	+	+	+	-	0	769		Unavailable			Unavailable			Unavailable	
50	Undetected		+	+	+	+	-	0	11830		Unavailable		-	0	1744		Unavailable	
51	Undetected		+	+	+	+	-	0	1043		Unavailable			Unavailable			Unavailable	
52	Undetected		+	+	+	+	-	0	1420		Unavailable			Unavailable			Unavailable	
53	Undetected		+	+	+	+	-	0	2342		Unavailable			Unavailable			Unavailable	
54	Undetected		+	+	+	+	-	0	1120		Unavailable			Unavailable			Unavailable	
55	Undetected		+	+	+	+	-	0	402		Unavailable			Unavailable			Unavailable	
56	Undetected		+	+	+	+	-	0	561		Unavailable			Unavailable			Unavailable	
57	Undetected		+	+	+	+	-	0	624		Unavailable			Unavailable			Unavailable	

Targeted deep sequencing:

The 36 genes targeted by NGS in peripheral blood DNA, buccal mucosa DNA, hair follicle DNA, and tumour DNA

Buccal swabs (N =11) and hair follicles (N =11) were collected from patients in which NF2 germline mutations were not identified by Sanger sequencing and MLPA. DNA was isolated from buccal swabs and hair follicles using DNA Extraction micro Kit (Qiagen). Tumour samples were stored at －80°C immediately after tumour resection during surgery. Tumour DNA (N=27) was obtained from frozen samples using DNA Extraction mini Kit (Qiagen).

We interrogated DNA derived from blood, tumour, buccal mucosa, and hair follicle to detect low VAF NF2 variants using HaloPlexHS targeted capture (Agilent Technologies, Santa Clara, USA). Targeted genes were NF2, SMARCB1, LZTR1, ALPK2, APC, ARID1A, ARID1B, BAP1, BRAF, BRCA2, CABIN1, CAST, CDKN2A, CDKN2B, COQ6, DDR1, EPB41L3, GLI1, MEN1, MUTYH, PIK3CA, PTCH1, PTEN, SDHD, SHH, SMARCE1, SMO, SUFU, TAB3, TERT, TP53, TRAF7, TSC1, TSC2, and WRN. Thirty-six targets comprising 724 regions were theoretically covered (Coverage rate: 97.97%) by 16733 amplicons of 176 bp average length for all coding exonic and intronic regions using Sure design (Agilent Technologies, Santa Clara, USA) as the custom design. The sequencing data were generated on the Illumina Hiseq 2000 sequencer.

Bioinformatics analysis and evaluation of variants

Data analysis of the number of reads for each amplicon in the HaloplexHS data was performed using the SureCall software V.3.0, provided by Agilent Technologies. Sequencing reads were aligned to the human genome (hg19). Sequence variants were identified using SureCall with calling parameters (minimum number of variant alleles ≥ 3, minimum sequencing depth ≥ 300x for SNPs, multiple nucleotide variations (MNVs), short indels, and VAF ≥0.1%). When an NF2 pathogenic variant (likely) was called using the SureCall, we re-checked the results of reads in the same position of NF2 gene in other organ DNAs even if the same NF2 variant was not called in that DNA.

Online supplementary Figure 1

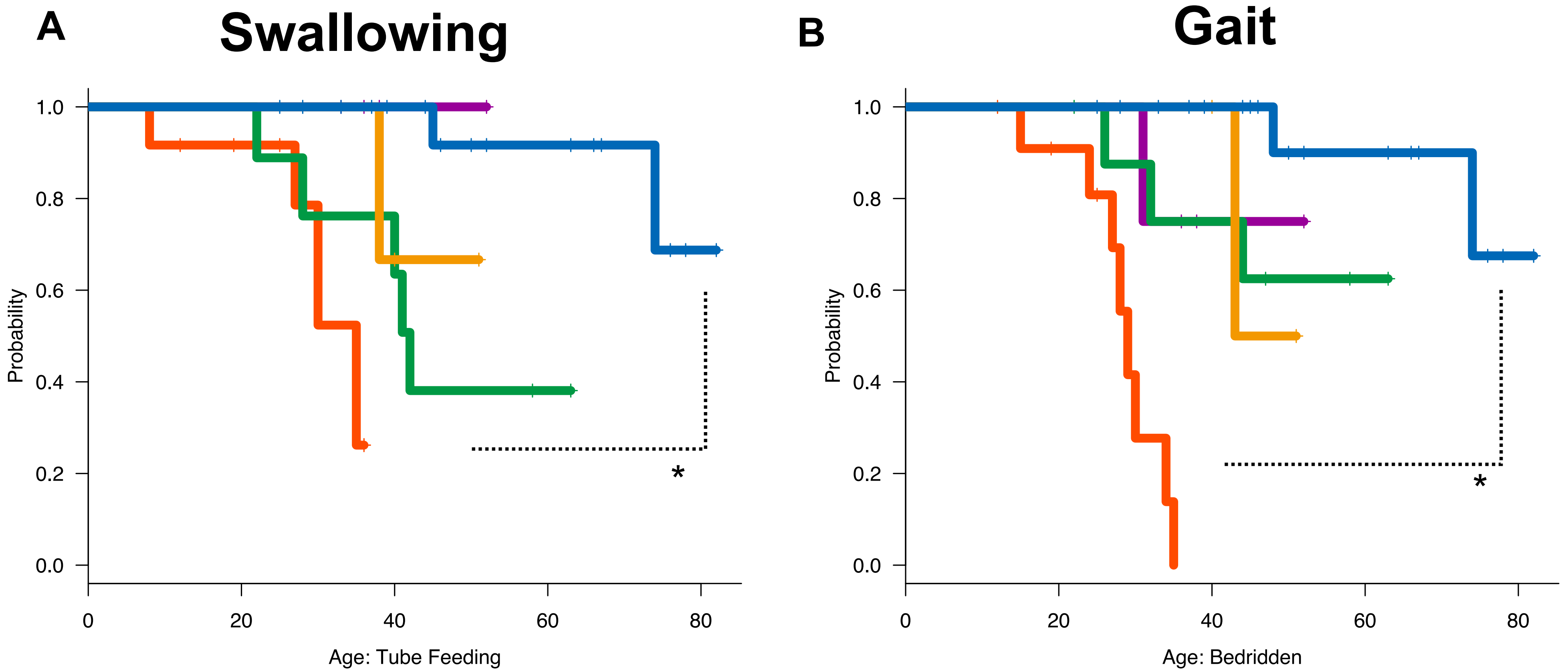

A Number at risk

	0	20	40	60	80
1.Truncating	12	8	0	0	0
2.Large del.	4	4	1	0	0
3.Splice site	9	9	6	2	0
4.Missense	3	3	2	0	0
5.Mosaic	20	20	13	7	1

B Number at risk

	0	20	40	60	80
1.Truncating	12	9	0	0	0
2.Large del.	4	4	1	0	0
3.Splice site	9	9	6	2	0
4.Missense	3	3	3	0	0
5.Mosaic	20	20	13	7	1

1.Truncating
2.Large del.
3.Splice site
4.Missense
5.Mosaic

Log–rank overall: $p < .0001$
Log-rank trend: $p < .001$

Multiple comparison
* $p < .0.01$

Online supplementary Figure 2

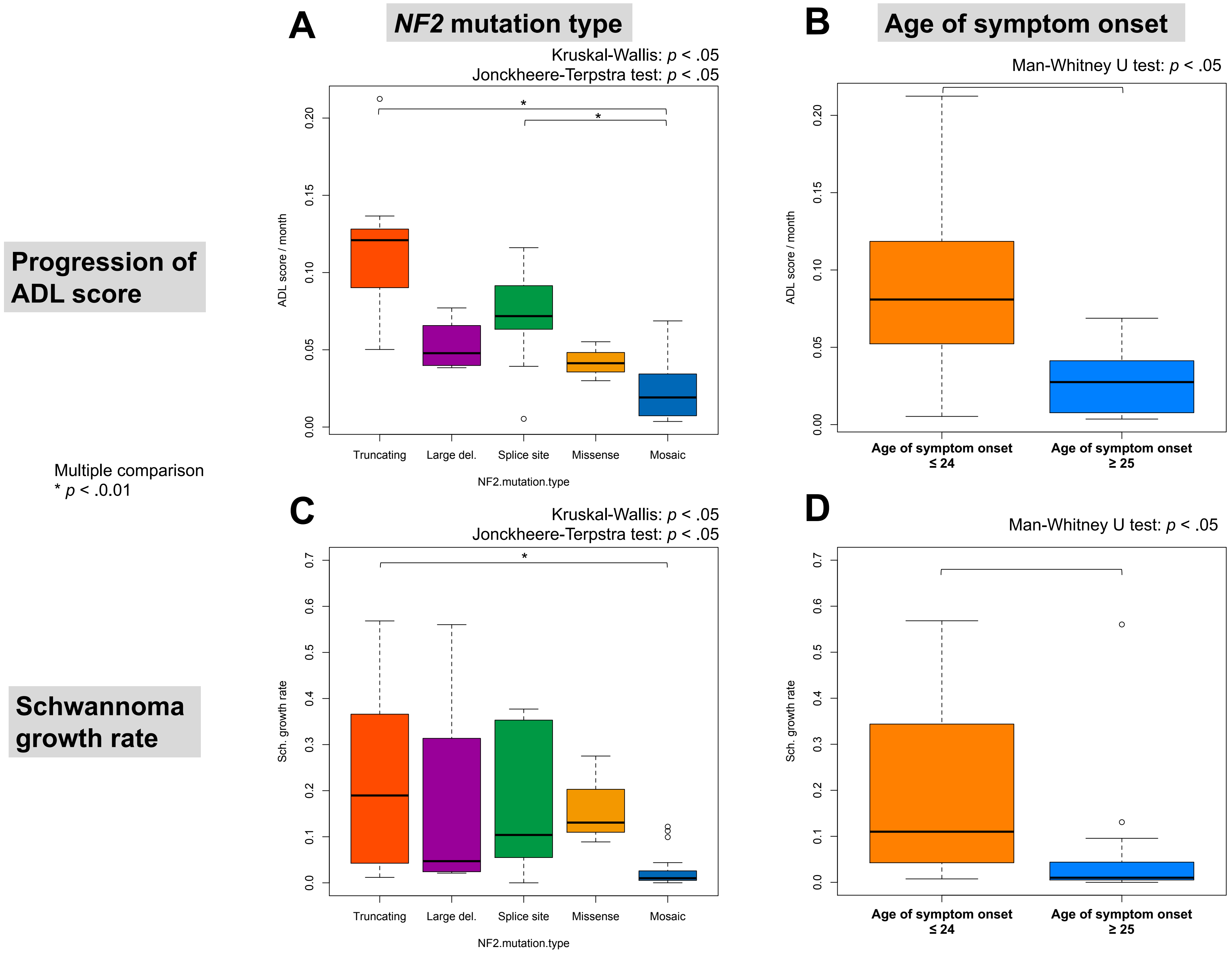

Online supplementary Figure 3

Applying previous genetic severity score (Halliday et al.) for our cohort

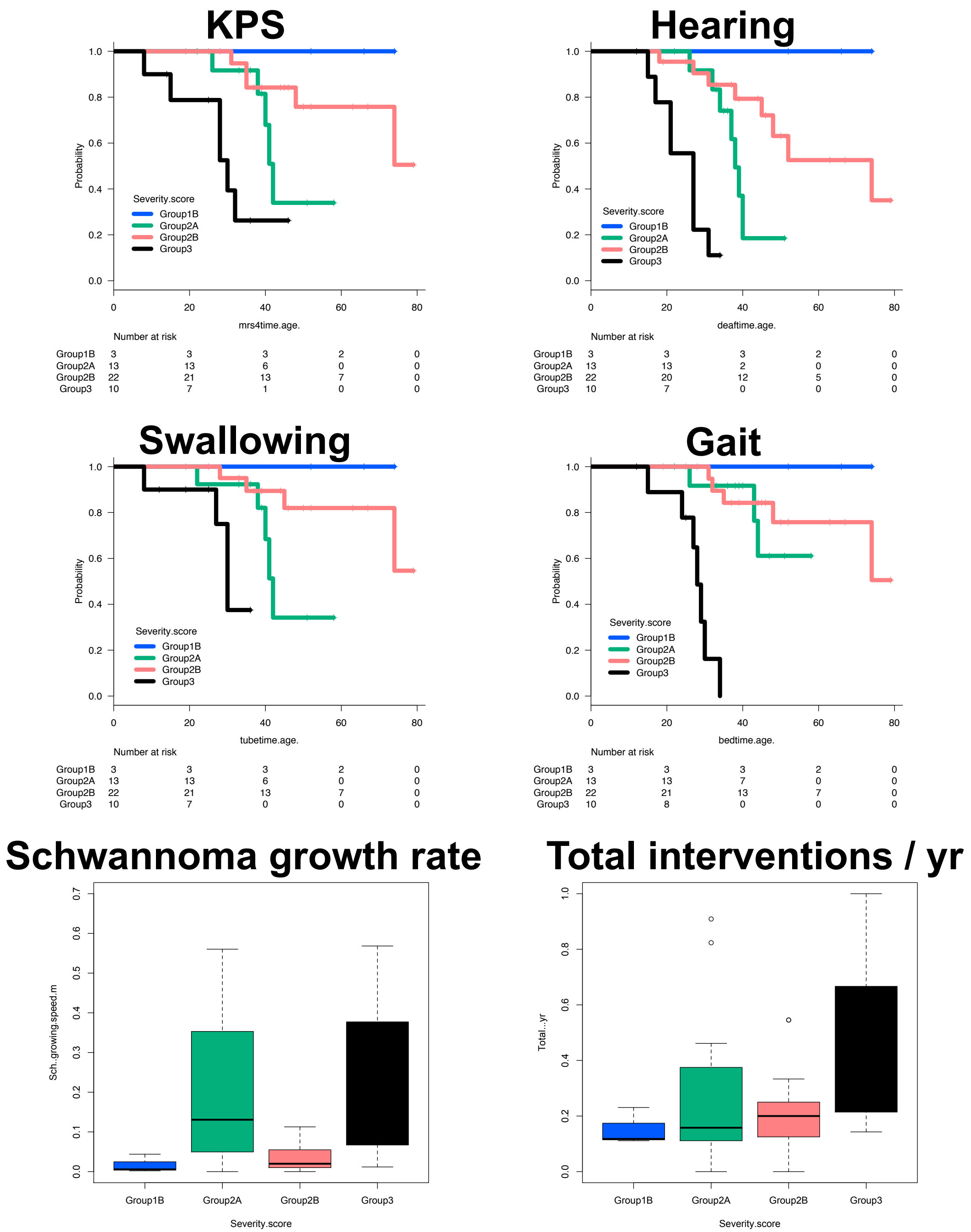

Supplementary Table 3. Genetic and clinical factor on KPS≦40 in NF2 patients

	<u>**Univariate Analysis**</u>	**HR**	**95%CI**	***p* value**
**Genetic predictors**	***Mutation type***			
	Truncating	10.81	3.55-32.86	0.00002716*
	Large deletion	0.91	0.12-6.94	0.9341
	Splice site	2.33	0.82-6.63	0.1123
	Missense	0.9	0.11-6.80	0.9187
	Mosaic, Undetected	0.11	0.03-0.39	0.0007178*
	Mutation location			
	Exon 2-7	2.41	0.93-6.23	0.06897
	Exon 8-13	1.56	0.58-4.18	0.3761
	Exon 1,14,15, Other	0.65	0.15-2.85	0.5728
**Clinical Predictors**	Onset age ≧25	0.03	0.005-0.29	0.001738*
	Onset symptom: hearing symptom	0.23	0.07-0.71	0.01068*
	Meningioma (+)	0.85	0.33-2.16	0.7346
	<u>**Multivariate Analysis**</u>	**HR**	**95%CI**	***p* value**
	Truncating	1.67	0.52-5.33	0.38
	Mosaic, Undetected	0.06	0.007-0.64	0.01*
	Onset age ≧25	0.01	0.0009-0.19	0.001*
	Onset symptom: hearing symptom	0.25	0.07-0.88	0.03*

* *p* value <0.05 was considered statistically significant.

Supplementary Table 4. Genetic and clinical factor on disabled hearing or deafness in NF2 patients

	<u>**Univariate Analysis**</u>	**HR**	**95%CI**	***p* value**
	NF2 Mutation type			
**Genetic predictors**	Truncating	15.26	5.00-46.53	0.000001672*
	Large deletion	0.60	0.08-4.49	0.6211
	Splice site	3.56	1.48-8.55	0.004511*
	Missense	1.36	0.31-5.87	0.6738
	Mosaic, Undetected	0.06	0.01-0.22	0.0000156*
	NF2 Mutation location			
	Exon 2-7	2.41	1.1-5.28	0.02787*
	Exon 8-13	1.218	0.52-2.8	0.6443
	Exon 1,14,15, Other	0.97	0.33-2.85	0.9699
**Clinical Predictors**	Onset age ≧25	0.16	0.06-0.41	0.0001654*
	Onset symptom: hearing symptom	0.39	0.17-0.90	0.02879*
	Meningioma (+)	0.95	0.42-2.1	0.911
	<u>**Multivariate Analysis**</u>	**HR**	**95%CI**	***p* value**
	Truncating	8.79	1.64-47.11	0.01*
	Splice site	3.30	0.79-13.72	0.09
	Mosaic, Undetected	0.08	0.01-0.51	0.007*
	Exon 2-7	0.74	0.29-1.87	0.53
	Onset age ≧25	0.10	0.02-0.39	0.0008*
	Onset symptom: hearing symptom	1.22	0.48-3.11	0.67

* *p* value <0.05 was considered statistically significant.

Supplementary Table 5. Genetic and clinical factor on tube feeding in NF2 patients

	<u>**Univariate Analysis**</u>	**HR**	**95%CI**	***p* value**
	NF2 Mutation type			
**Genetic predictors**	Truncating	18.2	3.14-105.5	0.001211*
	Large deletion	0.00	0-Inf	0.9985
	Splice site	3.168	1.03-9.72	0.04387*
	Missense	1.581	0.20-12.44	0.6633
	Mosaic, Undetected	0.13	0.03-0.48	0.002371*
	NF2 Mutation location			
	Exon 2-7	1.687	0.58-4.82	0.3296
	Exon 8-13	0.83	0.23-3.0	0.7885
	Exon 1,14,15, Other	0.90	0.20-4.05	0.8946
**Clinical Predictors**	Onset age ≧25	0.04	0.005-0.32	0.002528*
	Onset symptom: hearing symptom	0.33	0.10-1.06	0.06459
	Meningioma (+)	1.58	0.48-5.1	0.4443
	<u>**Multivariate Analysis**</u>	**HR**	**95%CI**	***p* value**
	Truncating	4.23	0.39-45.6	0.23
	Splice site	1.44	0.13-14.8	0.75
	Mosaic, Undetected	0.08	0.004-1.78	0.11
	Onset age ≧25	0.01	0.001-0.23	0.002*

* p value <0.05 was considered statistically significant.

Supplementary Table 6. Genetic and clinical factor on complete dependence or bedridden in NF2 patients

	**Univariate Analysis**	**HR**	**95%CI**	***p* value**
**Genetic predictors**	***NF2 Mutation type***			
	Truncating	31.33	7.82-125.4	0.000001124*
	Large deletion	0.97	0.12-7.51	0.9821
	Splice site	1.14	0.32-4.01	0.8367
	Missense	1.14	0.14-8.73	0.8997
	Mosaic, Undetected	0.11	0.03-0.42	0.001006*
	NF2 Mutation location			
	Exon 2-7	4.014	1.41-11.43	0.009247*
	Exon 8-13	1.24	0.43-3.53	0.686
	Exon 1,14,15, Other	0.30	0.04-2.31	0.2523
**Clinical Predictors**	Onset age ≧25	0.04	0.005-0.3	0.002003*
	Onset symptom: hearing symptom	0.11	0.02-0.49	0.00389*
	Meningioma (+)	0.9482	0.35-2.51	0.9148
	**Multivariate Analysis**	**HR**	**95%CI**	***p* value**
	Truncating	3.37	0.70-16.1	0.12
	Mosaic, Undetected	0.12	0.01-1.19	0.07
	Exon 2-7	1.31	0.34-4.99	0.68
	Onset age ≧25	0.02	0.001-0.30	0.004*
	Onset symptom: hearing symptom	0.23	0.04-1.16	0.07

* *p* value <0.05 was considered statistically significant.

Supplementary Table 7. The phenotype of patients with undetected mutation

Pt	Sex	Onset age	Follow -up	Germline mut. / Mosaic*	*LZTR1* mutation*	Vestibular schwannoma	Cranial nerve schwannoma	Spinal schwannoma	Multiple Meningioma	Extra CNS schwannoma	Skin/Orbit al lesion	Neuropathy
49	F	16	6	Undetected	Negative	+/+	-	-	-	-	-	-
50	F	21	24	Undetected	Negative	+/+	+/-	-	-	+	-	-
51	M	25	23	Undetected	Negative	+/+	+	+/-	+	-	-	-
52	M	24	23	Undetected	Negative	+/+	+	+/-	+	-	-	-
53	F	17	22	Undetected	Negative	+/+	+	+/-	+	-	-	-
54	F	59	5	Undetected	Negative	+/+	-	-	+	-	-	-
55	F	32	8	Undetected	Negative	+/-	-	-	+	-	-	-
56	F	22	5	Undetected	Negative	+/-	-	-	+	-	-	-
57	M	43	15	Undetected	Negative	+/-	+/-	+	-	-	-	-

*Whole-exome sequence
